# Tai Chi-based exercise program provided via telerehabilitation compared to home visits in a post-stroke population who have returned home without intensive rehabilitation: study protocol for a randomized, non-inferiority clinical trial

**DOI:** 10.1186/1745-6215-15-42

**Published:** 2014-01-30

**Authors:** Michel Tousignant, Hélène Corriveau, Dahlia Kairy, Katherine Berg, Marie-France Dubois, Sylvie Gosselin, Richard H Swartz, Jean-Martin Boulanger, Cynthia Danells

**Affiliations:** 1 Université de Sherbrooke, Sherbrooke, QC, Canada; 2 Research Center on Aging, University Institute of Geriatrics of Sherbrooke, Sherbrooke, QC, Canada; 3 Université de Montréal, Montreal, QC, Canada; 4 University of Toronto, Toronto, ON, Canada; 5 Sunnybrook Stroke Research Unit, Toronto, ON, Canada

**Keywords:** Balance, Stroke, Telerehabilitation, Tai Chi exercise program, Randomized controlled trial

## Abstract

**Background:**

The incidence of strokes in industrialized nations is on the rise, particularly in the older population. In Canada, a minority of individuals who have had a stroke actually receive intensive rehabilitation because most stroke patients do not have access to services or because their motor recovery was judged adequate to return home. Thus, there is a considerable need to organize home-based rehabilitation services for everyone who has had a stroke. To meet this demand, telerehabilitation, particularly from a service center to the patient’s home, is a promising alternative approach that can help improve access to rehabilitation services once patients are discharged home.

**Methods/Design:**

This non-inferiority study will include patients who have returned home post-stroke without requiring intensive rehabilitation. To be included in the study, participants will: 1) not be referred to an Intensive Functional Rehabilitation Unit, 2) have a Rankin score of 2 or 3, and 3) have a balance problem (Berg Balance Scale score between 46 and 54). Participants will be randomly assigned to either the teletreatment group or the home visits group. Except for the delivery mode, the intervention will be the same for both groups, that is, a personalized Tai Chi-based exercise program conducted by a trained physiotherapist (45-minute session twice a week for eight consecutive weeks). The main objective of this research is to test the non-inferiority of a Tai Chi-based exercise program provided via telerehabilitation compared to the same program provided in person at home in terms of effectiveness for retraining balance in individuals who have had a stroke but do not require intensive functional rehabilitation. The main outcome of this study is balance and mobility measured with the Community Balance and Mobility Scale. Secondary outcomes include physical and psychological capacities related to balance and mobility, participants’ quality of life, satisfaction with services received, and cost-effectiveness associated with the provision of both types of services.

**Study/trial registration:**

ClinicalTrials.gov: NCT01848080

## Background

The incidence of strokes in industrialized nations is on the rise, particularly in the older population. The risk of stroke doubles every ten years after age 55 [1]. The prognosis of functional recovery following a stroke depends on several factors, including initial severity of the injury, spontaneous recovery capacities and the impact of rehabilitation [2]. In older individuals, stroke sequelae can include sensorimotor, cognitive and perceptual impairments, which directly affect mobility and balance and thus drastically increase the risk of falls [3,4]. In fact, balance and ambulation problems are the most important fall risk factors following a stroke [5-7]. It has been shown that 75% of individuals who have had a stroke fall within the first six months following their discharge from hospital [3]. Consequently, balance is considered to be one of the most promising reversible risk factors to reduce falls [8,9].

In Canada, only 10 to 15% of people who have had a stroke actually receive rehabilitation services [10]. Typically, rehabilitation is based on a multidisciplinary approach that begins during acute care and continues in an intensive functional rehabilitation unit (IFRU) and after discharge. However, 37% of moderate-to-severe cases are transferred to a rehabilitation unit post-stroke [11]; the others return home with or without rehabilitation services [12,13], either because they live too far from a rehabilitation center or because their motor recovery was judged adequate for a safe return home [14,15]. Thus, there is a large number of individuals at home who need rehabilitation following their stroke but who do not have access to services [16]. Consequently, their mobility and balance decline, creating a significant risk for falls in this population.

The literature supports the need for outpatient rehabilitation services, at home or elsewhere for those who return home, regardless of the severity of their sequelae [17]. Unfortunately, the precarious nature of services has been well-documented in the Canadian Best Practice Recommendations for Stroke Care (updated 2013); it appears that the primary obstacle hindering best practice application in stroke care is the lack of human resources. With this in mind, the Best Practices Expert Consensus Panel prioritized certain recommendations, including facilitating outpatient and home-based rehabilitation services because of the inability of the healthcare system to meet current demands [18]. Therefore, it is essential to improve accessibility to rehabilitation services, as clearly indicated in the Quebec Homecare Support Policy [18,19].

In this context, new alternative approaches need to be considered to deliver services at home. Telerehabilitation, defined as a telehealth application that uses telecommunication technologies to provide rehabilitation services, is a new approach in the rehabilitation field. It has been identified as a very promising alternative tool that could help improve access to healthcare services in general [20-25]. Furthermore, telerehabilitation has been identified as one of the three telehealth application areas prioritized by the Quebec Ministry of Health and Social Services [11]. A main advantage of this intervention method is that it allows patients to receive rehabilitation in their homes without having to travel to get healthcare services. This way of delivering services also addresses the shortage of professionals; with less time spent traveling, professionals can spend more time treating, which in turn improves access to services. It is therefore crucial to provide decision-makers with evidence concerning the effectiveness of telerehabilitation applications.

Regardless of the rehabilitation delivery mode, the choice and type of exercise program varies from one study to the next. Recent studies have shown that a balance retraining program based on Tai Chi movements improved balance in individuals at risk of falls [26,27]. A Tai Chi-based exercise program, supervised by a physiotherapist, which uses movement repetition favoring directional adjustments in space has been shown to be effective in improving balance in individuals with physical impairments, including those presenting sequelae following a stroke. Two studies conducted by our research group demonstrated similar results in frail, older individuals [28,29]. A study conducted with older individuals with diabetes found that a simple sequence of Tai Chi movements improved their balance and attention capacity [29]. Another study conducted with frail, older individuals with balance problems showed that a Tai Chi intervention had a protective effect on the incidence of falls: for every person who had falls in the Tai Chi group, the conventional physiotherapy group had 1.3 people who had falls [29]. Furthermore, the Tai Chi intervention appeared to delay the onset of the first fall in older individuals, with 50% of participants in the physiotherapy group falling once after five months of follow-up, compared to after ten months of follow-up for 50% of participants in the Tai Chi group [30]. Even more interesting is the fact that these balance retraining programs based on Tai Chi movements are equally effective in older individuals following a stroke [31,32]. In fact, the first large-scale study [31] (Tai Chi group: n = 74; comparison group: n = 62) demonstrated the effectiveness of Tai Chi in improving balance compared to a conventional exercise program in individuals with chronic stroke. A second study [32] achieved the same results with 18 patients. Moreover, in a pre-/post- intervention study with no control group, 17 balance impaired elders have undergone a structured, interactive, and supervised Tai Chi class from their own homes through a videoconferencing system to improve balance and reduce fear of falling. The results demonstrated the intervention was effective for improving balance and reducing fear of falling [33]. Finally, our pilot study demonstrated that Tai Chi practiced via supervised telerehabilitation was effective in improving balance and motor skills in six patients post-stroke.

The principal aim of this clinical trial is to test the non-inferiority of a Tai Chi-based exercise program, provided via telerehabilitation compared to the same program provided in person at home, in terms of effectiveness for retraining balance in individuals who have had a stroke but do not require intensive functional rehabilitation. More specifically, the objectives are to:

1) Verify whether the gains in both physical and psychological capacities related to balance and mobility of patients treated by Tai Chi via telerehabilitation are not inferior to those receiving the same treatment in person at home;

2) Verify if the gain in quality of life of patients treated by Tai Chi via telerehabilitation is not inferior to the gain of those receiving the same treatment in person at home;

3) Verify if satisfaction with services received by patients treated by Tai Chi via telerehabilitation group is not inferior to satisfaction of those receiving the same treatment in person at home;

4) Compare the costs associated with the provision of the two types of services delivery, that is, via telerehabilitation and in person at home.

## Methods/Design

### Study design

The design of choice to determine the effectiveness of an experimental intervention compared to a standard intervention is the randomized controlled trial (RCT). Two groups will be formed: 1) telerehabilitation group, and 2) home visits group. We will use this design while considering that the prerequisites for undertaking a non-inferiority RCT are met:

1) The plausibility that a balance retraining program based on Tai Chi movements has been shown to improve balance [34], and specifically in clients with stroke [32,35].

2) The research procedures in telerehabilitation are well established in real-life situations, from both clinical and technological perspectives. Our team is at the forefront of conducting studies in home-based teletreatment following knee arthroplasty [30,36], in speech therapy [37], and in individuals with chronic obstructive pulmonary disease [38].

3) We conducted a pilot study in six patients post-stroke.

**Figure 1:**
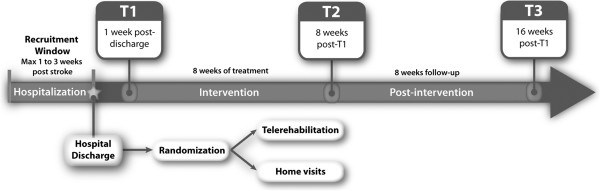
Research design and timeline.

Figure 1 illustrates the research design and timeline.

### Participants

The study population of interest will be individuals who have had a stroke and stayed in a hospital linked to one of the following sites: 1) Sherbrooke site (Centre Hospitalier Universitaire de Sherbrooke), 2) Montreal sites (Jewish Rehabilitation Hospital, Institut de Réadaptation Gingras-Lindsay-de-Montreal and Centre de Santé et de Services Sociaux Champlain-Charles-Le Moyne), and 3) Toronto site (Sunnybrook Health Sciences Center). Special care will be taken to ensure that participant eligibility will target individuals who have had a stroke and returned home without requiring intensive rehabilitation. We will base our selection on the Rankin classification [39] established by the treating neurologist or trained therapist during the hospitalization period. This classification system allows us to assign individuals who have had a stroke to one of four groups according to their level of functional impairment. Patients with a Rankin score of 2 generally present with mild impairments, maintain their autonomy and can perform all previous activities. Patients with a Rankin score of 3 present with moderate impairments and generally require moderate assistance with their activities of daily living. These patients are usually sent home with or without referral for physiotherapy services at home.

Therefore, a sample of participants aged 45 years and older will be recruited based on the following inclusion criteria: 1) have had a stroke with a Rankin score of 2 or 3; 2) not be referred to an IFRU and return home following discharge from hospital; 3) understand instructions to allow participation in evaluations and interventions; 4) have a balance problem as evidenced by a score between 46 and 54 on the Berg Balance Scale [40]; 5) have a caregiver who would be available during the telerehabilitation sessions to ensure safety during exercises; and 6) live in an area served by high speed Internet. Excluded will be individuals who present with: 1) a previous stroke episode in the last 12 months (other than the present one); 2) severe body hemineglect; 3) significant hemianopsia visual problems accompanied by hemineglect; 4) uncontrolled medical problems; and 5) moderate to severe aphasia.

Participants will be recruited during the hospitalization period by the team of treating nurses, physiotherapists and neurologists at each of the three sites (Sherbrooke, Montreal and Toronto). When the team decides that a patient will not require a referral to an IFRU and will return home (inclusion criteria 1 and 2), they will verify inclusion criteria 3 and 4 and exclusion criteria 1 to 5 from the medical chart. If patients are eligible based on these criteria, the hospital team will ask if they agree to be contacted by a research associate. If they agree, a research associate will visit them in hospital to inform them of the project and ask them to participate. The research associate will verify inclusion criteria 5 and 6. If a patient meets all the criteria and is interested in participating in the study, they will be asked to sign the consent form previously approved by the ethics committee at each recruitment site.

Once included, participants will be randomly assigned to either the telerehabilitation group or the home visits group by the coordinator of each recruitment site. This randomization will be performed using block randomization of sizes 2 and 4 done by a computer. Block size will not be known by the evaluators. This randomization will be performed following stratification based on the Rankin score (2 or 3), a variable that is likely to influence recovery prognosis. A system of numbered, sealed envelopes will be put in place.

### Ethics

The study is being conducted in accordance with the Helsinki Declaration. It was approved by the Ethics Committee of each recruitment sites: 1) Sherbrooke - Centre Hospitalier Universitaire de Sherbrooke and Centre de Santé et de Services Sociaux de l’Institut Universitaire de Gériatrie de Sherbrooke; 2) Montréal - Centre de Recherche Interdisciplinaire en Réadaptation de Montréal Métropolitain; 3) Longueuil - Centre de Santé et de Services Sociaux Champlain-Charles-Le Moyne; 4) Toronto - Sunnybrook Hospital. It has also been registered under http://www.clinicaltrials.gov (NCT01848080).

### Interventions

A personalized exercise program based on Tai Chi was developed by our team for previous studies aiming to improve balance in older individuals with diabetes [28] and in frail, older individuals with balance problems [29]. This program was also used during the pilot study. The exercise program consists of movements based on a combination of alignments and body-specific orientations, weight transfers and changes in direction inspired by Tai Chi [41]. The exercises do not require any physical interaction between the Tai Chi instructor and participants. Optimal treatment frequency for patients in the subacute and chronic phases following a stroke is twice a week, as suggested by a systematic review [42].

For both groups, the interventions will start immediately after discharge from hospital. The rehabilitation exercise sessions will be conducted by a physiotherapist trained in the practice of Tai Chi. The sessions will last 45 minutes and take place twice a week for eight weeks. The only difference between the groups will be the delivery mode of the intervention, that is telerehabilitation or home visits.

### Outcomes

All evaluations will be completed in the research centers/hospitals of the three sites and will last approximately three hours. The first evaluation will be conducted at baseline, prior to starting intervention (T_1_) to collect base measures on the participants’ condition. A second evaluation will be conducted just after the two-month intervention (T_2_) and the third evaluation will be four months (T_3_) following discharge from the hospital. All evaluations will be carried out by evaluators trained in the standard procedures for all measures, who are independent and blinded to the intervention. Specific directions will be provided to the evaluators and the participants to ensure that intervention allocation remains anonymous.

To measure balance and functional mobility, the Community Balance and Mobility Scale (CB&M) [43-45] will be used. This tool was developed for ambulatory individuals following a traumatic brain injury [43]. It has been validated in individuals following a stroke [44,46]. It assesses 19 tasks. The items are rated on a six-point scale (from 0 = unable to perform, to 5 = able to perform independently), for a maximum total score of 95 points. The CB&M has demonstrated good intra-rater, inter-rater and test-retest reliability (ICC = 0.98 for all three cases) and high internal consistency (Cronbach’s alpha = 0.96) [43]. It has good convergent validity with the Chedoke-McMaster Stroke Assessment [47] for leg and foot (r = 0.61 and 0.63, respectively) and for lower extremity strength (r = 0.67) [44]. Furthermore, the CB&M has been shown to be sensitive to change from baseline at follow-up in community-dwelling, ambulatory individuals post-stroke (SRM (standardized response mean) = 0.83) [44]. In addition, the Berg Balance Scale [42,48] will be used to evaluate balance in patients. The Four-Squares Test will also be used. This test consists of moving in different preset directions: forward, backward and to the sides. The time required to complete the sequence is recorded. An execution time greater than 15 seconds is predictive of falling. This test has high inter-rater (ICC = 0.99), intra-rater (ICC = 0.98) and test-retest (ICC = 0.98) reliability in community-dwelling older individuals aged 65 years and over [49]. Also, for predicting falls, this test has good specificity ranging from 88% to 100% and a positive predictive value of 86% [49].

The physical abilities capacities measured will be: 1) walking speed, 2) walking endurance, 3) lower extremity strength, and 6) lower extremity movement capacity. Each of these abilities plays an important role in achieving optimal functional mobility. Walking speed will be evaluated using the Timed Up and Go test (TUG) [50]. The test consists of standing up from a chair, walking a distance of three meters, turning around and returning to the chair to sit down again. The test is timed to measure walking speed in seconds over a distance of six meters. The TUG demonstrates good convergent construct validity with moderate to strong correlations with walking using the Tinetti test (r = 0.53) and walking speed (r = 0.66) in an elders population aged 65 years and over [51]. It has also been shown that walking speed is related to use of gait aids and fall frequency [52]. It is a measure sensitive to change [53]. Walking endurance will be estimated by the distance covered in meters during the Two-Minute Walk Test [53,54]. The inter-rater and intra-rater reliability of this test is high (ICC = 0.99) [50]. General lower extremity strength will be measured with the Sit to Stand test (STS) [55]. The time required to complete five successive ‘sit-to-stand’ repetitions will be recorded [55,56]. The test-retest reliability of the STS is high (ICC = 0.89) [57]. There is also a strong correlation of the STS with walking speed over five meters (r = -0.66) and lower extremity strength (r = 0.47) [58]. Lower extremity movement capacity and postural control will be evaluated using the Chedoke-McMaster Stroke Assessment [59]. The impairment inventory determines the presence and severity of physical deficits, and allows patients to be classified according to Brunnstrom’s seven stages of motor recovery. This tool has good intra- and inter-rater reliability (ICC = 0.98 and 0.97, respectively) and good construct validity (r > 0.60 with different aspects of the Fugl Meyer test) [47].

Two main aspects of psychological capacities measured will be 1) fear of falling, and 2) self-efficacy. The simplified version of the Activities-specific Balance Confidence (ABC) scale [60] questionnaire (ABC-S) will measure fear of falling. This questionnaire includes 15 questions rated on a four-point Likert scale and evaluates an individual’s balance confidence during a series of daily activities [60,61]. The ABC-S has strong internal consistency (Cronbach’s alpha = 0.86) good convergent validity with significant associations with balance and occurrence of falls [60]. Self-efficacy when faced with difficult life situations will be evaluated using the Generalized Self-Efficacy Scale (GSES) [60]. This questionnaire is a ten-item scale that uses a four-point Likert scale (from 1 = not true at all, to 4 = completely true) to assess whether an individual believes that their actions are responsible for their successful outcomes. This scale has been shown to have excellent internal consistency (Cronbach’s alpha = 0.86) [62].

Quality of life will be measured using the Reintegration to Normal Living Index (RNLI) [63]. This tool consists of 11 items ranked on a three-point Likert scale. The total score ranges from 0 to 22, with higher scores depicting lower quality of life. The psychometric properties range from very good to excellent with good internal consistency (Cronbach’s alpha = 0.90) and good test-retest reliability (r = 0.83) [63]. The construct validity of this tool has been examined in a post-stroke population and shows excellent correlations with the Frenchay Activities Index (r = 0.69) and the Short Form 36 Health Survey (r = 0.74) [64].

Participant satisfaction with the intervention received will be evaluated using the Health Care Satisfaction Questionnaire, a questionnaire developed and validated in French [65]. The satisfaction construct is determined by three distinct factors which include satisfaction with: 1) the therapist relationship, 2) the services provided, and 3) the organization of services. This tool includes 26 questions, scored on a four-point Likert scale. The total score is calculated based on average satisfaction for the three factors, with higher scores indicating higher levels of satisfaction. Test-retest reliability is documented by an ICC of 0.72 and internal consistency by a Cronbach’s alpha of 0.93 for the entire scale [65].

A modified version of the ‘Cost analysis of telemedicine’ table from the University of Minnesota [66] will help tabulate the costs associated with the teletreatments. These costs will be calculated to establish a cost differential (that is, cost-effectiveness) between the two different types of intervention. The method for collecting this data has already been tested in a previous study [67]. The variables related to the total cost include those associated with the actual intervention (for example, direct and indirect time for the intervention, professionals’ travel time to the participants’ homes, telerehabilitation equipment, and Internet service including installation and set-up).

The typical sociodemographic (for example, age, sex, marital status, living environment, education, primary occupation) and clinical characteristics (for example, diagnosis, gait aid, medications, self-rated health, co-morbidities) will be collected using an in-house questionnaire, with the exception of comorbidities for which the Functional Comorbidity Index will be used [68].

The level of impairment post-stroke will be measured by the National Institute of Health Stroke Score (NIHSS) [69,70]. This questionnaire, administered by a healthcare professional, includes 15 items to evaluate patients in the acute post-stroke phase. It demonstrates good internal consistency (Cronbach’s alpha > 0.5) [71], good inter-rater (ICC = 0.69) and test-retest (ICC = 0.66 to 0.77) reliability in a post-stroke population [69] and good convergent validity (r = 0.74 with lesion volume seven days post-stroke) [70]. The level of cognitive impairment will be measured using the Montreal Cognitive Assessment (MoCA) [72]. This test evaluates attention, concentration, executive functions, memory, language, visioconstructive capacities, abstraction capacities, calculation and orientation. The maximum score of 30 corresponds to no cognitive problems. The internal consistency of this test is good (Cronbach’s alpha = 0.83) and test-retest reliability is excellent (ICC = 0.92) [73]. The correlation between the MoCA and Mini-Mental State Examination scores has been shown to be excellent (r = 0.87) [73]. Noteworthy is the fact that this test will be completed during the participant recruitment phase.

### Statistical methods

We expect to recruit 240 participants, that is, 120 per group. Allowing for a drop-out rate of 10%, this sample size will provide a power of 80% to demonstrate the non-inferiority of Tai Chi provided by telerehabilitation compared to Tai Chi provided in person at home, with a two-group one-sided test at the 2.5% level (nQuery Advisor 7.0; Statistical Solutions, Boston, MA). For this calculation, the non-inferiority limit was set at four points on the CB&M scale and the standard deviation of the gain was estimated to be 10.4, based on findings from Knorr [44], since SRM = Mean_GAIN_/s_GAIN_ implies that:

$$S_{\mathrm{GAIN}}={\mathrm{Mean}}_{\mathrm{GAIN}}/\mathrm{SRM}=\left(51.3-42.7\right)/0.83$$

First, we will describe the characteristics of each group pre-intervention using averages and standard deviations (continuous variables) or percentages (categorical variables). The groups will then be compared using a *t*-test or chi-squared test. If the groups differ on certain characteristics pre-intervention despite randomization, subsequent analyses will account for these differences.

Primary analysis will aim to test the non-inferiority of Tai Chi provided via telerehabilitation compared to Tai Chi provided in person at home as evaluated by score gains on the CB&M questionnaire from T1 to T2. Delivery by telerehabilitation will be regarded as non-inferior to in person home delivery if the upper limit of the unilateral confidence interval around the difference in gains between the two groups is less than 4. This non-inferiority limit was set below the CB&M’s minimal clinically significant difference of five points. It is recommended to choose a non-inferiority limit less than the difference judged to be clinically significant [74,75].

The same analytical strategy will be used to evaluate and compare the effectiveness of the interventions on secondary variables and to compare maintenance over time (that is, the confidence interval around the difference between the two groups from T1 to T3) across all variables. A more conservative level of significance will be used to account for the numerous tests.

All analyses will first be performed according to the treatment received (per protocol = PP) and according to the assigned group (intention to treat = ITT), requiring that non-inferiority be shown in both cases since ITT analyses increase the chance of declaring non-inferiority if the two delivery modes produce a similar pattern of withdrawal, while PP analyses bias towards the null in presence of a different pattern of withdrawal [74]. For the ITT analysis, the effects of withdrawal or low levels of compliance will be explored with sensitivity analyses, where the missing data will be replaced with extreme values (that is, no change following treatment - the most favorable change noted in the study). Any non-robustness of results revealed by the comparison of strategies will be noted and the caveats will be mentioned in the discussion of results. All analyses will be conducted at the end of the study since the proposed treatments are of short duration and involve minimal risk to the participants.

The economic analysis will be a type of cost-minimization study [76]. Assuming that the effectiveness will be the same in the dependent variable (CB&M), the cost will be determined for the two groups and the cost differential will be established.

## Discussion

This trial is the first large-scale study to evaluate the non-inferiority of telerehabilitation versus home visits in patients with mild to moderate balance impairment post-stroke. Confirmation to recruit as many as 240 patients post-stroke with our inclusion/exclusion criteria is based on the 2010 to 2011 statistics for each recruitment site: 1) Centre Hospitalier Universitaire de Sherbrooke (269 participants admitted following a stroke, including 59 who returned home upon discharge); 2) Jewish Rehabilitation Hospital (277 referrals for patients post-stroke); 3) Centre de Santé et de Services Sociaux Champlain-Charles-Le Moyne (106 participants admitted following a stroke, including 60 who returned home upon discharge); 4) Toronto (189 participants admitted following a stroke, including 49 who returned home upon discharge). These well-documented statistics proved the realism of the scope of the study.

Telehealth, which includes telerehabilitation, is seen as a method to provide care to the population. Considering that the criteria for quality of care remain the same regardless of whether the care is provided by telehealth or other means, the evaluation framework of the proposed research is based on the theoretical construct of quality of care. Without formal definition [77], quality of care encompasses two components: 1) the viewpoint of the patient who considers the notions of accessibility and effectiveness as essential [78]; and 2) the viewpoint of the professional for whom the patient-doctor relationship [79], efficiency and reliability are of key importance. Several conceptual models for evaluating quality of care in the literature include the Donabedian [80], Total Quality Management [81], Quality-Caring [82], ORYX [80], HEDIS [83] and CONQUEST [80] models. In this research project, the conceptual evaluation model used will be the Donabedian model [84]. Given the objectives of this project, the study aims to answer specific questions related to the effectiveness of using telerehabilitation to improve quality of care for older individuals. This model defines quality of care as a combination of three elements: structure, process and outcome. The notions of ‘quality’ and ‘performance’ are directly related to the organizational structure of the healthcare system and its management processes. The term ‘outcome’ corresponds to the quality of care provided to patients, the performance of the healthcare system in offering care and the associated costs. Given the nature of this research, emphasis will be placed on the evaluation of telerehabilitation outcomes.

Concerning internal validity, we will control for potential selection bias by assigning participants to either the telerehabilitation or the control (home visits) group by randomization and by comparing participants’ characteristics in each group before the intervention. If the groups have different characteristics pre-intervention despite randomization, subsequent analysis will account for these differences. Information bias will be controlled by using standardized measures and by calibrating all the assessors for each assessment.

This study will verify the non-inferiority of in-home telerehabilitation compared to home visits for patients with mild to moderate balance problems post-stroke. Our hypothesis is that in-home telerehabilitation will be shown to be a good alternative to ensure continuity of rehabilitation services and their accessibility in the community.

## Trial status

Recruitment has just begun (September 2013).

## Abbreviations

ABC-S: Activities-specific Balance Confidence scale (Simplified version); CB&M: Community Balance and Mobility Scale; GSES: Generalized Self-Efficacy Scale; ICC: intra-class correlation; IFRU: Intensive Functional Rehabilitation Unit; ITT: intention to treat; MoCA: Montreal Cognitive Assessment; NIHSS: National Institute of Health Stroke Score; PP: per protocol; RCT: randomized controlled trial; RNLI: Reintegration to Normal Living Index; SRM: standardized response mean; STS: Sit to Stand test; TUG: Timed Up and Go test.

## Competing interests

The authors declare that they have no competing interests.

## Authors’ contributions

All authors participated in the conception and design of the study. MT is responsible for telerehabilitation expertise. HC and KB are responsible for fall prevention, balance expertise and intervention program. SG and RS are the neurologists taking care of recruitment. DK carries out the cost evaluation. MFD carries out the statistical analysis. JMB and CD supervised the team of research agents at their sites. All authors read and approved the final manuscript.
